# The Prevention of the Earlier Stages of Dental Caries

**Published:** 1911-07-15

**Authors:** Stanley Colyer


### THE PREVENTION OF THE EARLIER STAGES OF DENTAL CARIES.

BY STANLEY COLYER, M.D. (Lond.), M.R.C.P.

The multiplicity of causes, which have at one time or another been advanced to explain the origin of this disease, very largely fall into the class of predisposing or contributing causes which, although important, are yet not essential, whilst a few may be considered as the actual or exciting causes without which the disease cannot exist.

In this essay it will be our endeavor to trace, as far as lies within our power, the changes which have taken place either in man, or in his environment, or in both, which have rendered his teeth liable to caries, and to consider those changes with a view of ascertaining how far they can either be improved or removed so as to lead to a decreasing incidence and possibly a final disappearance of this most disastrous disease.

### GENERAL CONSIDERATIONS.

The first great advance towards solving the mystery of this disease was the discovery by Magitot that it was an external disease dependent upon an acid, the origin of which he did not correctly determine. The second great advance was made by Miller, who, so far as we are able to judge, correctly described its pathology. This work of Miller has been the means of leading to the discovery of an aetiology of sufficient accuracy upon which to base a successful prophylaxis. The growth of this aetiology and prophylaxis has been gradual, and only during the last few years has evidence of its success been sufficiently apparent to change the outlook of the ultimate control of this disease from one of extreme pessimism to one of comparative optimism.

The reason for this most unfortunate delay would appear to have been due to the fact that most workers had studied caries in its later stages, after cavity formation, and omitted the far more important work of investigating it in its earliest. In 1904, S. Colyer published a paper, which bore upon this important point.* This observer noticed that in normal mouths the earliest stage of caries occurred as a rule in two well-defined regions, namely, at and around the points of contact of the teeth and in the fissures, and that it did not occur at points where solid food usually lodged, namely, at the necks of the teeth. The uniformity of position and the character of the lesions suggested that they were due to an acid fluid held against them by capillary attraction, and that the acid fluid, if the pathology of the disease were correct, was formed from a solution of soluble and fermentable carbohydrate. It may be added that fissures and pits in teeth are capillary spaces, and although it is not here argued that therefore caries in them is due to fermented fluid, it is probable that it is as important a factor as solid food.

It is difficult to overestimate, especially from the point of view of prevention, the importance of realizing that caries may be due to fermentation in a fluid, but we must not for that reason be led to under-estimate the part played by solid food, more especially in those mouths with misplaced teeth. Solid food would appear to act in three ways. Firstly, as a block or obstruction to the free flow of saliva between the teeth and into the fissures, which thus prevents the natural dilution and carrying away of soluble carbohydrate or acid solution. Secondly, as a sponge in which an acid fermentation and a growth of micro-organisms is taking place, and which would act as a source of supply of acid, carbohydrate or organisms to the fluid held between the teeth. And thirdly, as a pabulum for increasing the number of acid-producing organisms in the mouth.

---

*"The problem of Dental Caries," by S. Colyer, *Dental Record*, July 1904.

THE FACTORS CONCERNED IN THE CAUSATION OF CARIES.

It is now generally agreed that four factors are together concerned in the production of dental caries, namely, the tooth, the saliva, the micro-organisms and the food.

(1) THE TOOTH.

The clinical evidence that in approximal caries the adjacent teeth are often acted upon in a different degree suggests that they possess different degrees of resistance—a very interesting fact when it is remembered that the points at which adjacent teeth touch are not, *with the exception of the upper central incisors and the lower central incisors, calcified at identical periods of life.** The recent experiments of Stanley Mummery on the resistance of different teeth to a solution of lactic acid of known strength bears out this clinical deduction. Mummery used a solution containing 0.75 per cent. of lactic acid and in it bathed the teeth under observation. He calculated their resistance to caries by the difference in time they took to be affected, and he found remarkable differences.† It would seem that the question whether a tooth will become carious depends, partly upon its molecular structure, as suggested by Miller, partly upon the *degree of concentration* of the acid, and partly upon the *time that the acid is allowed to act.* The inference from this is, that although it would not be safe to assume that any form of enamel is absolutely immune to caries, yet it is highly probable that the degree of acidity which would, in a given time, produce caries in one would be unable to do so in another. We can only consider therefore, the word "immune" as applied to teeth in a comparative and not in an absolute sense. Its importance will be realized if it be remembered that the causes which lead to the de-

---

*"Dental Surgery and Pathology," 3rd edition. By J. F. Colyer.

†*Proc. of Roy Soc. Med.* Feb., 1910. "Some experiments on the relative susceptibility of different teeth to caries." Stanley P. Mummery.

struction of enamel act *intermittently and during varying lengths of time.* As it is probable that the most resistant teeth are, as a rule, found in the mouths of those physically most perfect, it behoves us to examine for a moment those factors which give rise to healthy human beings. They fall naturally into two classes, the congenital and the environmental.

*By congenital* we mean the natural biological tendency of the fertilized ovum to develop along certain lines. On the whole a type tends to breed true, a good stock giving rise to a good type and a poor to a poor type, and congenital peculiarities to be reproduced in certain members of a family. The popular opinion that certain families are more liable to caries than others is probably correct, and may be dependent partly upon the structure of the tooth, partly upon peculiarities of the saliva, and partly upon family habits. Selective breeding from families showing immunity to caries would tend to produce the immune type. This certainly offers one method of aiding in the prevention of the disease, but, unfortunately, it is one from which little or no hope can be anticipated at the present day.

*By environment* we mean all those influences that may be brought to bear upon a human entity from the moment it begins to receive nourishment from its mother to the moment of the complete formation of its teeth. Apart from congenital tendencies the people that have the best formed teeth are those whose environment has been of the most suitable nature. Clearly, then, it is important that the health of the child's mother during its prenatal life should be good, and that its postnatal feeding should be correct. It is now well established that, physically speaking, a child is best served if its mother, being able to do so, nourish it. The normal period of suckling in civilized communities is considered to be nine months, and this period is gradually being reduced to six. In less civilized countries, or those that have been less under Western influence, the period is in some cases three years, for example, in

Japan and among the aborigines of Australia. It would therefore appear that the duration of suckling to-day is but a third, or even less than that given to it by our prehistoric ancestors. This decrease in breast-feeding in itself would tend to produce a tooth different in structure from that of our ancestors, but whether of better or of inferior structure it is impossible to say. During and after the process of weaning the child lies at the mercy of its caretakers, and many a one has been launched on to the world a physical wreck owing to the ignorance of "nurses and the prejudiced counsel of grandmothers." Dr. Forsyth has recently shown that in England in Elizabethian times the recognized period of infant suckling was three years; that this period was gradually reduced during Tudor and Georgian times, and is to-day considered to be eight months. The effects of this extraordinarily rapid change in the environment of the infant which has thus been brought about is worthy of very close study by medical men.—*British Medical Journal,* April 15, 1911. The full recognition of the importance of correct infant feeding renders it unnecessary to more than draw attention to it, and to insist that defective feeding does unquestionably lead to teeth of defective formation and of increased liability to caries. Closely related to this aspect of our subject is the suggested influence of lime contained in drinking water on the structure of teeth. It has been maintained that in districts in which lime is in small quantities is absent from the water, the people are more liable to caries. This is the reason given by some for the condition of the teeth of the people of Glasgow. Rose, by comparing different districts, endeavored to show that such was the case in Germany. The result of Rose's painstaking work were published in 1894, and although they at first sight appear to prove his claim, they are so full of statistical fallacies that too much importance must not be put upon them.*

---

*"On the decay of teeth in the National Schools," by C. Rose. ***Oesterreichisch-Ungarische Vierteljahrsschrift fur Zahnheilkunde,*** 1894.

### (2) THE SALIVA.

Researches into the *role* that saliva plays in dental caries have yielded for the most part results of a negative character. The reaction of saliva is said to be normally alkaline, but it is often amphoteric, and frequently acid. Alkaline saliva appears to favour the growth of mouth organisms, whilst acid has a slight tendency to retard them. Alkaline saliva neutralizes the acid formed by fermentation, and might by that means preserve a tooth if it could thus prevent the presence of free acid sufficiently long to allow fresh supplies of saliva to diffuse into and slowly remove the fermenting mass. This interesting supposition is borne out by Rose, who examined the saliva of 219 children, of an average age of 13 years, and concluded that an alkaline saliva constituted the best means of checking caries in its early stages.* Miller also found that no free acid was formed in equine saliva, which is strongly alkaline, when incubated with a carbohydrate solution under twenty-four hours. In view of this experimental evidence it is somewhat surprising to note that J. F. Colyer found dental caries present in 66 horses out of a total of 484 examined.†

In children in whom rickets develops towards the end of the first year and after the eruption of the lower four and upper two incisors, it will often be found that these teeth are completely decalcified, and little more or less than brown leathery masses of dentine are left, the original contour of which is preserved, no proteolytic digestion of their colloidal substance having taken place. Such is obviously not a caries in the usually accepted sense of the word. We examined several of these children and found, as we expected, saliva of a remarkably acid nature. This observation, unimportant as it may seem, is useful in that it shows the effect of bathing teeth in an acid medium, namely, com-

---

**Deutsche Monatsschrift fur Zahnheilkunde.* Dec., 1905.

†*Transactions Odontological Society.* Vol. 38, p. 42.

plete decalcification, and would appear to us to effectually dispose of the theory that the common form of dental caries is due to an acid condition of the oral secretions.

Rickets is a disease of early life, and it is not known whether its influence may have a permanent effect upon lowering the alkalinity of the saliva; but it is possible that this factor, chiefly supposed to lie in the food, may persist and, although incapable of producing acid saliva may reduce its alkalinity. Rose found that a diet consisting of food stuffs rich in calcium salts increased the alkalinity and quantity of saliva.

It is probable that saliva plays no small part in caries, but our knowledge of it at the moment is insufficient to turn it to practical advantage.

### (3) THE MICRO-ORGANISMS.

Dental caries is not a specific disease in the sense that it is dependent upon a specific micro-organism, or specific combination of organisms. Any organism which is capable of living in the mouth and of leading to an acid fermentation in a carbo-hydrate medium, *or any acid-producing organism which is capable of living for a while upon any carbohydrate medium with which it has been introduced,* is a potential agent for the destruction of enamel and consequently the induction of caries. There are experimental reasons for believing that the number, nature and kind of these acid-producing organisms depend very largely upon their environment, that for example, in a mouth in which a residuum of carbohydrate is frequently left they increase in number and possibly in intensity; that, in a mouth in which the residuum is generally of a proteid nature, they decrease, and, coincidentally, there is an increase of putrefactive organisms. It is by no means uncommon to come across subjects whose mouths throughout life have been innocent of any form of oral hygeine, and yet who show no signs of caries. In them the teeth and the surrounding tissue will generally be found covered with a slimy mass of decomposing mucous and epithelial *debris*, which fills the interdental spaces and coronal fissures, and so blocks those positions where carbohydrate food is wont to collect. Moreover, in them, as Goadby and Miller have shown, the putrefactive organisms have obtained the upperhand. It has even been stated by Goadby that he has been able to produce excellent results in certain patients in whom caries was progressing rapidly by judiciously sewing the spores of bacillus mesentericus about their mouths and at the same time withholding carbohydrates.(*a*) Such a treatment is undoubtedly ingenious, but its application is scarcely simple enough to render it generally applicable. Moreover, it would seem to us that the frequent use of mouth lotions containing some soluble proteid, such as the caseinogen of milk, would, providing carbohydrates were withheld, bring about equally satisfactory results.

The means by which acid-producing organisms find access to the mouth will be dealt with under the next section.

### (4) THE FOOD.

As one looks down the vista of time to the unknown moment, a quarter of a million years or more ago, when the homo-simian of the family of anthropoids acquired reason of a nature so peculiar that it was to raise him to an immeasurable height above his associates, one realizes the utter impossibility of judging with any correctness his habits and subsequent evolution. We can learn directly but little of him, and must turn of necessity to existing types, both animal and human, and infer from their customs and life the changes that may have taken place in his. He did not enter at once into a garden of Eden, a garden planted with delectable and luscious fruits, a land flowing with milk and honey. He found an uncultivated land, and, taking from

(*a*) "The buccal secretions and dental caries," by Kenneth Goadby. ***Brit. Med. Journ.***, 17 Sept., 1910.

experience such of it as he desired by selective cultivation, he determined in a large measure the land products that to-day primitive man is found surrounded with. How many thousand years elapsed before he learned to shape the weapons he used in the chase, how many more before he learned to cook? It is supposed that a hunting and fishing period preceded that of cookery and that during it man passed through an age of increasing carnivorism. The introduction of cooking brought within his diet new sources of vegetable food hitherto too indigestable and in some cases poisonous and unpleasant, and probably led to an increased consumption of vegetable food and a proportionate falling off of animal food.

That caries was extremely rare in ancient times is unquestionable. Green examined the skulls of 26 cliff-dwellers who lived in Arizona and Colorado, some 5,000 years ago, and found only one instance.(*c*) Wright examined certain skulls in the Driffield Museum. Of 47, brought originally from the East Riding, belonging to the Neolithic and Bronze age, he found only one case, but four teeth showed signs of having been abscessed.(*d*) Mummery examined 68 skulls from "long barrows," and found two with caries; 32 from "round barrows," and found seven with caries. Of 23 skulls of Romans found at York he found 18 with caries. Of 76 Anglo-Saxon skulls 15.8 had carious teeth.* In this country it appears that there was a gradual increase of caries during the luxurious time of the Romans, and that it was followed by a well-marked decrease after their departure, when the country was overrun by the more barbaric Angles and Saxons who swept away for the most part the customs and habits of the Romans and introduced those of their less civilized land. Since the Anglo-Saxon period

---

(*c*) ***Items of Interest***, 1895.

(*d*) "The teeth and jaws of a series of prehistoric skulls," by William Wright. ***Journ. Brit. Dent. Assoc.***, Feb., 1903.

*"On the relation which Dental Caries, as discovered among the ancient inhabitants of Britain, and amongst existing aboriginal races may be supposed to hold to their food and social condition." ***Trans. Odonto. Soc.*** Vol. 2.

there has been a fairly steady increase until the commencement of last century, when the rate, especially during the latter half, rapidly increased, and reached its maximum to-day.

The introduction of cooking brought into man's diet food of an entirely different nature, namely, soluble starchy and partly dextrinized carbohydrates (and thus more easily fermentable), and we find as a consequence the first beginnings of caries. Since the discovery of tropical countries there has been introduced into this country, and indeed into all European countries, a food that hitherto was only obtainable in fruits and in the form of honey, namely, sugar. The Romans in their degenerate days were people of advanced epicurean tastes. They indulged largely in honey and heavily sweetened wines, and we are, therefore, not surprised to find that Mummery found in the skulls he examined such a high degree of caries. The height to which the cultivation of honey had come in the time of the Romans will be realized by anyone who cares to read the Fourth book of the Georgics, which is given up entirely to apiculture.†

It is well-known that the high-class Egyptian in the days of prosperity of their country suffered terribly from this disease. Professor Elliot Smith and his assistants examined several thousand Egyptian skulls and found that in predynastic and early dynastic times (until 2700 B. C.) caries was exceedingly uncommon, but that after that date, the time of the pyramid builders, it suddenly became so common that amongst the remains of 500 (mostly aristocratic) people of this date only about 50 were free. Since then the disease has gradually spread to all classes.(*a*) The food of the Egyptians is well known and its study from the point of view of caries is instructive. Bread in Egypt was made from a "kind of millet, like the modern dhurra, barley and rarely wheat. The grains were rolled and crushed

---

†"The Georgics," Book IV,. by Virgil.

(*a*) *Brit. Med. Journ.*, page 622; 3 Sept., 1910.

on a stone and then both the flour and *the bran* were mixed with water into a stiff paste; from this pieces were broken off and flattened out by the hand into cakes of various degrees of thickness which were baked on hot stoves, or in mudlined ovens." The bread cakes were made in a variety of shapes, flat, round, oval and fancy. Wheat, which was a luxury in Palestine and in Egypt, appears to have been a food chiefly of the better classes; partly it would seem because it would make a lighter and a softer bread owing to the presence in wheaten flour of a large amount of gluten. The bread was leavened and there can be little doubt (for reasons which we will show later) that its acidity was often high. But apart from this cause for caries among the aristocratic Egyptians there were other and important ones in operation. Thus sour milks or curds were often consumed which would not only introduce acid into the mouth but also acid-producing organisms. We read also that not only the baker but "the confectioner found constant employment in every town and village in Egypt, for the Egyptians loved cakes made with honey and fruit of all kinds and bread and buns made into fanciful shape." Beer made from barley and variously flavoured was the common beer of the country; but there was another and sweet beer made from honey. Figs and dates (each contain about 50 per cent. of glucose) were common fruits.(*b*)

We turn now to a more detailed description of certain articles of food.

*Sugar.* In England in the middle ages sugar was restricted to the upper and wealthy classes, and even at the beginning of last century its price was prohibitive except to the well-to-do. Even so early as the reign of Queen Elizabeth its use had become somewhat prevalent, and a belief that it was a cause of bad teeth was evidently current; for Heutzer, a German traveler, in describing, as an eye-witness, the manner in which the great Elizabeth went

(*b*) "A Guide to the Egyptian Collections in the British Museum," by E. A. Wallis Budge.

to Chapel at Greenwich, says of her appearance that "her teeth were black (a defect the English seem subject to from their too great use of sugar)."(*c*)

There are certain people who do not take sugar, either on account of a personal antipathy or for other reasons. It will be found, as a rule, that they are free from caries. These antipathetic cases are rare; we have only met with two, one of which was reported to us. In both cases: of two children of the same family, one ate sweets and the other did not; the former suffered and the latter was free from caries. Sim Wallace records a somewhat parallel case in which a child of eight years had perfect teeth, whilst a younger child of the same family had caries more or less advanced in four of her teeth. The younger child was fond of and ate liberally of sweets, the elder had never been allowed to eat them as they were supposed to have given rise to a skin eruption in early life.(*d*) Another valuable clinical contribution of a similar nature is contained in a paper by Warwick Hele. He records the case of three sisters who were of a precise and careful nature; "In each and all caries was so rapidly progressive that before the eldest girl had attained her majority almost all her teeth had been filled, many in several places, and especially on approximal surfaces. The second girl was no better off, and the third girl, then about seventeen, was considerably worse off." The youngest was then sent to Germany for three years and during that time she had no reason to visit a dentist, and merely followed as was her custom the usual oral hygiene. On her return not a single tooth required filling; but after she had been home for twelve months her teeth became even worse than her sisters'. Hele gives as his explanation that the whole family was addicted to sweets *et ad hoc genus omne*. Abroad she had been unable to obtain supplies and had perforce to abstain from indulging her taste, but on her return, in her own words,

(*c*) "The Town," by Leigh Hunt.
(*d*) "Cause and Prevention of Dental Caries," by Sim Wallace, 1906.

"she ate all she could get to make up for lost time."(*a*)

Ottofy, in a paper on "The teeth of the Igorots," brings out the relation of sugar to caries, though not absolutely conclusively. In the Igorots, a semi-barbaric race, he found 2.05 per cent. of carious teeth. The diet of these people consists of food requiring considerable mastication; bread is unknown and sugar distasteful to the children. In the Filipinos, the more civilized inhabitants of the islands, there was 20.9 per cent. of carious teeth. These indulge freely in the practice of chewing sugar which is sold in small pieces all over the islands.(*b*)

The fact that many of the natives working on sugar plantations have good teeth is due, we believe, partly to the comparative resistance to fermentation of raw sugar, and partly, and chiefly, to the habits of the individuals. But such is not always the case, as is apparent from a paper by D. S. Ricard. He writes of the sugar plantations in Natal which are worked by coolie labour imported for the purpose from India: "The principal ailment these people suffer from is toothache. The young cane is very luscious; it is also very nourishing. The food the coolie lives on is not rich in health sustaining ingredients; it consists principally of rice, oil, dhole and ghee, so the people make up the deficiency by chewing cane continuously. 'I can tell you how long a man has been on the estate by his teeth,' said the overseer, to the writer, 'that is to say,' he corrected himself, 'any time up to two years; after two years the teeth get into such a state that they can deteriorate no further, as far as outward appearances go, at any rate."(*c*)

Let us repeat once again that sugar, except in the form of honey and fruits, is of comparatively modern introduction into civilized countries. The great increase of caries during last century would appear to have occurred coin-

---

(*a*) "Dental Fillings: a cause of their failure, and remedy," by Warwick Hele. ***Brit. Dent. Journ.***, 1903.

(*b*) ***Dental Cosmos,*** July, 1908.

(*c*) "Sugar and Toothache," by D. S. Ricard; ***Dental Record,*** July, 1904.

cidently with its increasing introduction. In 1905 the consumption of sugar *per capita* in Great Britain was 77.83 lbs., in the United States 92.46 lbs., in New Zealand 108.64 lbs. Pickerill, of Otago, in mentioning these statistics, gives it as his opinion that the incidence of dental caries in these countries is roughly in the same proportion.*

During last century glucose, a mono-saccharide, manufactured artificially by heating with dilute mineral acids the poly-saccharides—sucrose, lactose, maltose, starch and gums—has been used in increasing quantities. The cost of its production is small. So long ago as 1885 it could be produced at ½d. per pound. Its use is wide. As an adulterant to cane sugar it can be added in large proportion without altering the appearance. It is used in the manufacture of table syrups, candies, toffees, artificial honey preserved fruits, jams and marmalade—in fact in most forms of sweet produce, and more especially those in which crystallization is to be avoided. A sugar of such universal use must, if the saccharoses are dangerous, to the teeth be infinitely more so. Goadby states, "As a general thing it may be said that it takes about 2½ times as long to ferment a given quantity of cane sugar by means of mouth bacteria, as it does the same amount of glucose or maltose. In the majority of instances a 2 per cent. solution of glucose or maltose in peptonic water having a reaction neutral to litmus will show an acid reaction in five or six hours, often less, cane sugar requiring about 36 hours." These results are interesting and show the harm one may expect from mono-saccharides and especially in the form of sweets as eaten by poor children. "With a limited pocket and an unlimited desire for sweets, the poor child affects chiefly those which have the greatest lasting property, such for example, as jujubes, stick-jaws, gums, caramels, and so forth; in other words, the less soluble and those containing the largest quantity of gummy material. The process of

*"The Medical Aspect of Dentistry and the Necessity of Dental Instruction for Medical Students," by H. P. Pickerill. *Brit Med. Jour.*, 13 Feb., 1909.

sucking is often a long one, at times extending over several hours, and not infrequently only completed as the child falls asleep. Imagine, for one moment, the condition of things in this latter case: the whole mouth is covered with a thick glutinous covering of glucose, the saliva ceases to flow, the movements of the tongue cease, and then, perhaps, for nine or ten hours the micro-organisms are given a clear field for their efforts. In adults, and in older children, the conditions are somewhat different. In them, if sweets are sucked long enough for the saccharine fluid to collect between the teeth, it may be hours before the saliva is able to diffuse into and dilute these portions sufficiently to render them harmless; moreover, they are at spots *inaccessible to the tooth brush* and dislodgeable only with difficulty and patience by swilling fluid about the mouth." Does this not explain why caries occurs at points of greatest capillary attraction, even in those patients who, though using a tooth brush at night, fail to complete the cleansing by swilling out the mouth with water or some other innocuous fluid?

*Bread.* Though we believe sugar in its various forms of by far the most important cause of the caries of to-day there are many other possible ones. Of these perhaps the principal is bread. It has been urged that the flour which is made from roller mills, which in this country have since 1879 been gradually replacing the old stone mills, is the cause of the enormous increase of dental caries in modern times. One of the most prominent advocates of this view is T. G. Read, who, in the early part of the late nineties, conducted some experiments concerning the action during mastication of roller and stone-milled flour. He states that he discovered an increase of 40 percent. during mastication in the acidity of bread produced from roller-milled compared to that produced from stone-milled flour. The question was reinvestigated by J. B. Knight, an analytical chemist, who reported "that the acidity in the bread made from stone-milled flour increases to a very slight extent in the process of mastication—not more than would be accounted for by the acidity of the mouth, but with the bread made from roller-milled flour there is an increase of 40 per cent. after mastication."* From these statements based upon a very few experiments many dental surgeons have come to have a strong antipathy to roller-milled flour; and it must be admitted that, without a careful consideration of them, they are extremely alarming. Let us examine the evidence. It will be observed that neither the nature nor the amount of the acid is stated. If the acidity formed during mastication—and it is almost impossible to believe that such acidity was in any way due to the bread being masticated—from stone-milled-flour-made bread can be accounted for by the acidity of the mouth, which as a rule is generally alkaline, it must have been very slight, and an increase of 40 per cent. found in the roller-milled-flour-made bread could not have been very great. If the experiments of Stanley Mummery can safely be applied to changes taking place in the mouth, we should not expect such a slight degree of acidity to do much harm to the teeth in, for instance, twenty-four hours. Further, there is no evidence that the bread was baked by the same baker, or whether yeast or baking powder was used. It has been suggested that the explanation lies in the fact that there are less lime salts left in roller-milled flour than in stone-milled to neutralize the acids—chiefly succinic—that are normally formed during the "spongeing" of bread. There is nothing either in the composition or the manufacture of these flours to account for this difference in acidity of the breads in question, and we have, after considering the matter, come to the conclusion that the reason must lie in outside causes. Putting aside the possibility of the acidity being due to a badly compounded baking powder, as this substance is

---

*"An address on the importation of flour," by T. G. Read, 1902.

rarely used in the manufacture of bread, we believe that the explanation will be found in the yeast.†

Yeast nearly always contains lactic acid-producing organisms. Brewers aim at a pure yeast because the presence of the organisms is apt to sour their beer soon after manufacture. But bakers are not so particular, and the yeast they use often contains considerable numbers of adventitious germs. That many of these are not destroyed in the process of baking is evidenced by the fact that bread, especially if at all moist, may sour. Now, in the old days, brewers' yeast was generally used to bring about the condition of "spongeing," but in recent years this has given place to many new methods of which the following are the most important:—

*Scotch barms.* About forty years ago a substance known as Parisian "barm" was introduced into Scotland. "Barm" is essentially a "leavened ferment"—a scientific modification of a system followed in ancient Egypt—made by mixing and fermenting together malt, hops, yeast and flour. It differs from ordinary bakers' yeast in containing four, which thus makes it into a "leaven". After its introduction it spread rapidly and was soon used by most of the breadmakers of Scotland and many in the North of Ireland. Scotch "barm" always shows a certain proportion of lactic ferments as a normal constituent; *their growth being definitely encouraged.* As a consequence Scotch bread always has a slightly acid flavour, resembling in type the flavour of buttermilk (Jago).

*Compressed yeast.* Owing to the difficulty of obtaining brewers' yeasts or of manufacturing home-made yeasts, the use of compressed yeasts has in recent years become com-

---

†Since writing this essay I have found that Jago has carefully investigated the causes of the acidity of bread. He finds that modern flour is crowded with adventitious organisms and believes that this is a more important cause than the presence of the lactic acid organisms in many yeasts. It would certainly be interesting to discover how these organisms gain access to the flour, and further whether more are found in the roller-milled product than in the stone-milled. Such a discovery might lead to an invention for their exclusion—an event that would certainly be welcomed by bakers, if not by dental surgeons. Those interested in this aspect of the subject will find it discussed fully in Jago's "Science and Art of Breadmaking."

mon in Great Britain and elsewhere. These are imported into this country, chiefly from France, the Netherlands and Germany. They are yeasts used in the production of spirits from malt and raw grain. It is well known that distillers often allow their malt to lie until lactic acid organisms have developed in considerable numbers on the grain, as the subsequent fermentation is consequently more energetic and complete, resulting in a larger yield of spirit. This statement does not apply to distillers whose principal object is the production of yeast. The yeast is skimmed from the fermenting liquor and allowed to settle; it is then partly dried by compression, and mixed with a small proportion of starch. Such compressed yeasts vary greatly; some are almost pure, others contain foreign bacteria in large numbers.

*Baking powders.* These consist as a rule of a mixture of tartaric acid and bicarbonate of soda. In some cases acid phosphate of soda is used, and rarely bisulphate of potash and ammonium bicarbonate.

*The aeration process.* In the original method, invented by Dr. Dauglish, ordinary aerated water was mixed with the dough under pressure; the aeration occuring when the mixed product was removed from the kneading vessel. This process has now been modified. A weak wort, made by mashing malt and flour, is allowed to ferment until it has become sour, and some of this is then mixed with the water to be aerated. It is claimed that by this improved process more carbon-dioxide is absorbed and that the acid softens the gluten (Jago).

The acidity of bread is a well-recognized fact. Notter and Firth state that it generally averages from 4.5 to 6 grains per pound. They consider that 8 grains per pound (.114 per cent.) ought certainly to be the limit. The acidity is stated in terms of acetic acid.* Whitelegge and Newman note that in making bread a certain proportion of dextrin

*"Theory and Practice of Hygiene," by Notter & Firth.

and sugar is formed from starch and that traces of lactic and butyric acids may be found.†

In alcoholic fermentation the acidic by-products are acetic, butyric and succinic acids; of these succinic occurs in the largest amount. The lactic acid found in bread must either be present in the original yeast or be formed from the diastased starch during the process of baking and cooling. Baking occupies an hour to an hour and a half; and it is probable that during that time the interior of the loaf never rises above 212°-216°F., and that the interior of the loaf only remains at its maximum temperature for a very short while, so that there is good reason to believe that many sporeing varieties of lactic acid organisms are not killed. In proof of this is the well-known condition of sour bread when induced by the slow cooling. Jago relates an interesting example in support of this view. Four loaves, fresh from the oven, were brought into an office. One of these was taken at once into an inefficiently ventilated room containing three or four hundred people. After remaining two and a half hours in this room it was broken and emitted a decidedly sour odor. The other three, which had been left in the office in cool air, were next morning still perfectly fresh and sweet. Jago, in giving advice for the prevention of acidity, remarks: "Acidity is favoured by the use of weak, unhealthy yeast, unstable flours used with excess of water, fermentation being conducted at too high a temperature, insufficient baking, and cooling in a warm, impure atmosphere."

Apart from bread, there are other common articles of food of a slightly acid nature which contain lactic acid-producing organisms. We refer to butter, cheese, clotted cream and milk.

*Butter* may be made from (1) sweet cream, (2) sour or ripened cream, (3) ripened whole milk, or (4) clotted cream.

---

†"Hygiene and Public Health," by Whitelegge & Newman.

Readers interested in the question of the purity of flour are referred to reports Nos. Cd 5613 and 5614 (Price 1d. each), recently issued by the Local Government Board.

In dairies where churning only takes place once or twice a week, butter is always made from sour milk. Souring or ripening is brought about in two ways known as the (1) natural method, or (2) that of 'starters.' In the former the milk is allowed to stand for three or four days at 60°F. When it is ready for churning it contains about .5 per cent. lactic acid. In the latter method, 'starters,' that is powders containing lactic acid organisms are added to separated milk, which the following day is added the whole milk and left for twenty-four hours. A portion of this is then mixed with the cream to be used for churning. When the butter has "come," it is usually washed twice to get rid of the casein. At this stage the butter, which is granular, is soaked in brine for twenty-four hours, then placed on a worker, allowed to drain for 15-20 minutes and then "worked" into a solid mass.

*Cheese* is made in a somewhat similar manner. It is only necessary to point out here that in none of the so-called "scaldings" which are part of the process does the temperature rise above 130°F. (most of them never rise above 100°F.)

*Devonshire cream* is made as follows: milk is allowed to settle for twelve hours in the summer and twenty-four in the winter, in circular pans about seven inches deep. These are placed over a hot water stove, and heated so that in half an hour they have attained a temperature of 175°-180°F.—this is the scalding process. They are then cooled quickly, as quickness improves the keeping qualities. When cold the cream, which is thick and clotted, is skimmed off.

*Ordinary milk*, when first drawn, is amphoteric in reaction, but rapidly becomes acid, owing to the action of adventitious organisms. When consumed it is always slightly acid and loaded with acid-producing and other organisms.

### SUMMARY OF THE PRINCIPAL CHANGES THAT HAVE OCCURRED IN FOOD.

Of the changes that have taken place in man's diet since the early days of his origin, the most important, we believe, are the following:—

(1) A gradually increasing amount of soluble and fermentable starchy carbohydrates.

(2) An increasing amount of sugar, and in recent years especially a marked increase of the more easily fermentable mono-saccharides.

(3) A gradual elimination of the harder portion of food

(4) An increasing number of articles of food in a partly. acidly fermented state, which not only constantly introduce acid, but also acid-producing organisms. The acidity of the food would tend to precipitate the mucous and some of the salts of the saliva, and to neutralize its alkalinity.

Of these changes the two most important probably are, the increased amount of soluble fermentable carbohydrates, and the constant introduction into the mouth of acid-fermenting organisms.

### THE PREVENTION OF DENTAL CARIES.

Only through a complete knowledge of the causes of caries can we hope to discover the means of complete prevention. To-day our knowledge is incomplete, so that we are unable to formulate with any certainty the rules for absolute prevention by natural means; but we can deduce from our knowledge certain methods for both natural and artificial prevention, which, if carefully followed, will, we believe, lead to a remarkable diminution of the malady. For the purpose of clearness we will divide the methods into:—

(1) Natural.

(2) Artificial.

(3) Indirect.

(1) *Natural methods.* In the first place every effort should be made to produce normal human beings. The feeble and the diseased should be discouraged from marriage and if married from having children. Mothers should be encouraged to suckle their children (indeed we would compel the able to do so by law), and the pulpy, pultaceous diet of the child after weaning should give place to one requiring more use of the jaws. It should be remembered that in suckling, a baby has to use considerable effort, so that when the time for change of food arrives it is ready for solid food. Care should be taken to include plenty of proteid in the food. The condition of the child should be watched and regulated. No infant should be allowed to become fat, as this is merely evidence of incorrect use of the digestive organs and often of inefficient digestion. By judicious care the child will thus grow up with good quality teeth and well-formed jaws, and the crowding of the teeth —an important predisposing cause of caries—be avoided.

In ordinary life, the order of food in meals should be carefully regulated and every effort made to leave the mouth clean and free, or comparatively free, from fermentable carbohydrate. Such results can be brought about by ending a meal with a proteid or fatty substance, by means of nuts, apple or other fresh fruit, as recommended by Wallace. If such means were followed it is probable that in many cases it would be unnecessary to do any thing further. But we must remember that the possibility of ending meals with fresh fruit is not open to the vast majority of people, who are poor, and the best we can recommend them with regard to diet is to eat as hard food as possible, and not to take anything of a fermentable carbohydrate nature, such as bread and jam, for at least two hours before going to bed and to forbid all sweets at night time.

(2) *Artificial methods.* Whilst we believe that a large decrease in caries could be brought about by an alteration in the diet of the races and should certainly be aimed at, yet we realize that such a change would take many years to accomplish. In the meanwhile, we have other methods upon which we can place much value. The one out-standing fact in the aetiology of the disease is that clean teeth do not decay. We believe that if extreme care were taken by every individual to bring about this state of cleanliness immediately after every meal, the disease would cease to exist. But such a method would be too exacting to most people and would not be generally adopted. If artificial prevention is to be successful among the generality of people it must fulfill three conditions: firstly, it must be simple; secondly, it must not disturb in any marked degree the existing routine of life; thirdly, it must be applicable at suitable times of the day. It is for these three reasons that if it were absolutely necessary to clean the teeth after every meal, we could hope for little immediate improvement. We have given it as our opinion in a previous part of this essay that, except in cases of patients who have teeth exceedingly susceptible to the influence of small quantities of acids, in other words, who have very susceptible teeth, the early stage of caries occurs as a rule at night, when the functions of the mouth are in abeyance and the organisms are then given ample time to bring about their lamentable results. We therefore believe, and we know it to be correct in many cases, that failing the more ideal method of cleaning the teeth after every meal, most excellent results will be produced by cleaning them at night time only and by taking no food of a fermentable carbohydrate nature, such as biscuits, sweets or milk, afterwards.

The method of cleaning teeth is of extreme importance, and it is on account of the absence of certain details in it that the tooth brush has come into such a bad repute. A small hard tooth brush, in which the bristles are of different lengths, should be used. The brush should be applied to all surfaces of the teeth, and its action may be aided by a mixture of chalk and Castile soap, although this is not essential. After the brushing the mouth should be thoroughly swilled out with pure water. The brush clears away the solid *debris*, the water dilutes and carries away the dissolved carbohydrate held between the teeth and in deep fissures on crown surfaces, by capillary attraction. *The second part of the process is absolutely essential, as it cleans*

*that portion of the teeth which the brush fails to reach.* Cleaning teeth in the morning is a social duty, but is not necessary as a means of preventing decay.

*Indirect methods.* We divide these into (*a*) Educational, (*b*) Administrative.

(*a*) *Educational.* We know that an instinctive knowledge of prevention is not common and that chiefly by means of education do people learn. What means then can we adopt to diffuse this important information? The teaching of hygiene, including oral hygiene, in all schools, must be made compulsory. It is a matter of indifference whether such instruction be specialized in certain teachers or not. We are of the opinion that no teacher's education should be considered sufficient until he has obtained a grasp of the rules for leading a healthy life. The information conveyed to the scholars should be simple, dogmatic and easily remembered. No harm is done to a child by instilling into its mind a generous conceit of its personal well-being and appearance; and it should be impressed with the awful importance of the fact that in maintaining its body in a healthy state it is not only doing a duty to itself and to the whole nation, but also a duty to its Maker.

We can expect little from a child except under direction, and if we fail to obtain the intelligent co-operation of those who have the home-control of the child the teaching at school will be of little avail. The parents must be educated. Is it possible to educate them? There are two important ways. A systematic crusade in the form of popular lectures should be given throughout the country by recognized authorities on the subject. The lecturers should either be members of the staffs of hospitals or paid and authorized persons. This is one method. The other is more important and more direct; it is by means of school medical officers. Medical inspection is to-day nearly universal throughout the country. At a very large proportion of these inspections one of the parents is present. It is from the advice given by the school doctors at these meetings that the greatest benefit that will assuredly accrue from medical inspection is to be expected. It would appear to us advisable that the knowledge of dental subjects in these officers should be of a uniform nature, and in order to bring such a result about, local authorities before appointing them should require evidence that they have had some special opportunity of gaining a knowledge of dental diseases. Such would be a method of dealing with growing generations of public elementary school children. An effort should be made on the part of dental authorities, to whom the rest of the profession look for an opinion, to diffuse a definite method of prevention and thus give strength to their suggestion by unanimity of opinion.

We wish now to refer to other methods of still more indirect nature. There can be no question in the minds of those who have studied the ultimate effects of decay of teeth and other dental diseases on the body that their influence is well marked. It seems certain that oral sepsis is an important factor in leading to secondary infections such as septicaemia, malignant endocarditis, septic gastritis, that it seriously handicaps recovery from such illnesses as typhoid and scarlet fevers, pneumonia and pernicious anaemia, that prolonged absorption of the septic material may induce peculiar states of lowered vitality of the body and open the gates to the ingress of other diseases; in a word, that it is liable to influence in many ways the duration of man's life. We would therefore advise that medical officers for life insurance should be induced to take this subject into consideration when reporting upon the value of the life of a client, and either advise their office to refuse the life until the source of danger has been removed, or suggest an advance in the premium usually demanded. In the same way medical officers holding appointments in connection with health societies giving sick pay should be approached. Such official recognition would very rapidly give rise to a sense of the importance of the teeth in the heads of families throughout the kingdom and produce in them the feeling that prevention is worthy of consideration.

(*b*) *Administrative measures.* The possibilities that we here refer to are such as can be carried out apart from the individual—passive measures. They will depend for their existence upon the discovery of the causes, or of important contributory causes, over which the individual has practically no control. As an example, let us take the case of typhoid fever. This disease, as is well known, may in some cases be water-borne, in others milk-borne. Preventive medicine has stepped in and by administrative measures has prevented the pollution of water and of milk, and thus removed largely two very important means of disseminating the disease. We have advanced the theory in this essay that a possible source of caries may lie in the constant infection of the mouth both by acid-producing organisms and of acids by means of bread, milk and, in a less measure, by butter, cheese and acidly fermented substances. If these substances are ultimately found to play an important part there can be no reason why they should be allowed to be consumed if manufactured in such a dangerous state. Administrative control could then step in and regulate the methods of maufacture with a view to producing these substances in a less harmful state. Such a discovery would be the ideal method of prevention and one that all interested in the subject must whole-heartedly hope for.

For our own part, we look forward with every belief in the ultimate conquest of this disease. We see a future people free of it, and marvelling that those of to-day could have suffered so long and so patiently. We imagine them asking themselves what strange perversity could have filled the mind of a people who were willing to spend thousands, nay millions, of pounds a year on the cure of a disease, upon discovering the cause of which they would not spend a penny. Can we wonder at them if they think thus? For everywhere we see the signs of this dreadful malady; it ruins the beauty of many, it enfeebles those who would be otherwise healthy, it helps to fill the workhouses and poorlaw infirmaries with preventible sickness, it is the direct and indirect cause of a waste of fabulous sums of money. And yet there is no one who will come forward, either a government or a charitable individual, and say, "There is a sum of money; try and rid the world of this terrible calamity."—*Dental Record* for May and June 1911.

